# Exploring Healthy Ageing in Place for Roma Communities: Insights from Knowledge Café Workshops in the UK

**DOI:** 10.1007/s10823-026-09561-4

**Published:** 2026-03-17

**Authors:** Ryan Woolrych, Aleksandar Marinov, Judith Sixsmith, Margaret Greenfields, Rosemary Cisneros, Petr Torak, Ann Hyde, Crina Morteanu, Gaba Smolinska-Poffley

**Affiliations:** 1 Heriot-Watt University, Edinburgh, UK; 2 University of Dundee, Dundee, UK; 3 Anglia Ruskin University, Cambridge, UK; 4 Coventry University, Coventry, UK; 5 COMPAS Charity, Peterborough, UK; 6 Community Renewal Trust, Glasgow, UK; 7 Luton Roma Trust, Luton, UK; 8 Roma Support Group, London, UK

**Keywords:** Roma communities, healthy ageing, place-based approaches, ageing-in-place, United Kingdom

## Abstract

Healthy ageing is increasingly understood as a process shaped by intersecting structural, social and environmental conditions. Yet dominant frameworks often fail to account for how marginalisation and place-based exclusion are lived and navigated by structurally excluded groups. This is especially true for older Roma communities, who remain significantly overlooked in both research and policy on ageing. Drawing on qualitative data from Knowledge Café workshops held in Govanhill (Glasgow, Scotland), Luton and Peterborough (England), this paper explores how place, identity and inequity shape experiences of healthy ageing within Roma communities. These workshops brought together policymakers and practitioners alongside Roma community members and advocacy groups. Using reflexive thematic analysis, we identify four key themes: (1) mistrust of health services and linguistic exclusion; (2) the symbolic and practical dimensions of ‘home’ in supporting ageing-in-place; (3) gendered and spatialised experiences of social isolation; and (4) the significance of community-led and culturally competent initiatives. Our findings highlight the critical importance of locally embedded responses that support culturally meaningful pathways to healthy ageing. We argue for a fundamental reframing of healthy ageing policy, toward co-produced, place-based and equity-driven approaches that recognise intersecting inequalities and value the lived expertise of marginalised older populations.

## Introduction: Ageing, Inequity and the Importance of Place in Roma Communities

Healthy ageing is widely understood as a multidimensional process shaped not only by individual capacities, but also by the social, spatial and structural conditions that enable or constrain wellbeing in later life. These include access to supportive environments, culturally responsive healthcare, secure housing and meaningful social relationships (Menassa et al., 2023; WHO, 2020; Wiles et al., 2012). Central to this understanding is the recognition that the places in which people age, whether homes, neighbourhoods, or community spaces, profoundly influence wellbeing, autonomy and dignity in later life (Buffel & Phillipson, 2024; Woolrych et al., 2022). Recent policy frameworks have placed growing emphasis on ‘ageing-in-place,’ which promotes the ability of older people to remain in familiar settings, supported by local infrastructure and social networks (Pani-Harreman et al., 2021). While these frameworks have advanced the ageing agenda, they often overlook the lived realities of older adults in marginalised or underserved communities, and rest on normative assumptions of stable housing, digital access, local social capital and culturally competent service provision, conditions rarely met by many minoritised groups (Quigley et al., 2022; Waugh & Mackenzie, 2011).

Roma populations represent the single largest ethnic minority grouping in Europe, estimated to comprise between 10 and 12 million people (EC, 2020). Rooted in centuries of discrimination and socio-economic exclusion, Roma represent one of the most marginalised and institutionally excluded groups across the continent (FRA, 2022). Yet, older Roma adults remain largely absent from academic discourse and ageing-related policy frameworks, despite the decade that has passed since the *Declaration on Healthy Ageing of Roma People* (WHO, 2014). For Roma individuals living with disabilities, a reality for many in later life, the extent of social exclusion is even more profound (European Disability Forum, 2022). Social determinants of health such as income, housing, education, employment and healthcare access are systematically and unequally distributed, with Roma communities consistently faring worse than majority populations across nearly all indicators (Cook et al., 2013; FRA, 2022). These inequalities are cumulative and shaped across the life course, contributing to the premature onset of age-related conditions and limited access to care in later life (Orton et al., 2019; WHO, 2014).

In this paper, we follow UK usage of the term ‘Roma’, which refers specifically to people of Roma origin who have migrated to the UK from Central and Eastern Europe (and their descendants), rather than to UK Gypsy or Traveller populations. This differs from the broader European policy definition, where ‘Roma’ is used as an umbrella term encompassing Roma, Sinti, Kale, Manouche, Romanichal and various Traveller groups. Our focus is therefore on non-UK-born Roma residing in the UK and their families, reflecting the migration-driven Roma populations present there. 

Although migration to the UK following the post-2004 wave of EU accession altered the social and material conditions of life for many Roma individuals and families, it has not resolved long-standing patterns of exclusion. Roma communities continue to face multiple intersecting barriers to participation, including in education, employment, civic life and access to public services (Rogers, 2021; Scullion & Brown, 2016). These experiences, shaped by a history of pre-migration exclusion, are compounded by frequent encounters with discrimination, bureaucratic gatekeeping and limited access to culturally appropriate care and communication, contributing to widespread mistrust in health and welfare institutions (Alexiadou, 2023; Kostka et al., 2024; McFadden et al., 2018). These barriers become particularly acute for individuals with low literacy in the official language of their country of residence or in written Romani dialects. European evidence also suggests that Roma women may face additional restrictions on mobility, privacy, or voice in healthcare encounters, intensifying their marginalisation (Földes & Covaci, 2012; Mózes et al., 2024; Plaza del Pino et al., 2022). Such exclusions go beyond isolated service gaps to reflect entrenched systemic patterns in which healthcare environments are not designed to accommodate the specific needs, histories or social realities of Roma populations (EPHA, 2020; Janevic et al., 2011).

At the same time, there is evidence of significant resilience and resourcefulness within Roma communities. Informal care networks and cultural norms of interdependence can serve as critical sources of support (y Blasco & Fotta, 2023; Greenfields, 2017; Málovics et al., 2019). Community-led models such as Roma health mediators, outreach hubs and integrated support services, have shown promise in addressing healthcare access barriers, improving trust and delivering more responsive services (Bueno, 2023; Greenfields & Ryder, 2012; Szilvasi & Saitovic-Jovanovic, 2023). However, despite their potential effectiveness, these approaches often suffer from short-term funding arrangements and remain underutilised. Research into how such approaches might support healthy ageing in Roma populations is limited and under-explored.

In sum, while there is a growing body of research examining the relationships between ageing, home and place among minoritised ethnic groups (Yazdanpanahi & Woolrych, 2023; Zubair & Norris, 2015), and across diverse urban contexts marked by social and spatial inequality (Buffel, 2017; Woolrych et al., 2021), Roma populations remain significantly underrepresented in these debates. Addressing this gap, this paper explores the intersections of healthy ageing and place amongst Roma communities in the UK.

Focusing on three case study sites - Govanhill (Glasgow, Scotland), Luton, and Peterborough (England) - where significant Roma populations reside, the paper explores how older Roma individuals make sense of ageing, identify barriers to accessing support and articulate culturally grounded visions of ageing in place. Based on participatory Knowledge Café workshops, the analysis examines the spatial dimensions of inequality, the symbolic and material importance of ‘home’ and the role of place in shaping understandings of healthy ageing. In doing so, the paper argues for a rethinking of ageing through the lens of equity and place justice (Hayes et al., 2025; Miranda et al., 2019), recognising both the socio-spatial constraints that shape ageing for Roma individuals and the collective strategies that offer pathways toward more inclusive ageing interventions.

## Methodology

### Context

This study draws on qualitative data from a series of Knowledge Café workshops held in three urban areas of the UK with established Roma communities: Govanhill (Glasgow, Scotland), Luton, and Peterborough (England). Govanhill is a densely populated inner-city neighbourhood in the south of Glasgow which contains one of the largest concentrations of Central and Eastern European Roma residents in Scotland. All Roma community members who participated in the Glasgow-based workshop lived within this neighbourhood, reflecting the concentrated pattern of Roma settlement in the city, which is why we refer specifically to ‘Govanhill’ throughout the paper rather than to Glasgow more generally. In contrast, Roma communities in Luton and Peterborough were recruited from a number of deprived wards where Roma households are known to cluster and where local partners identified significant levels of socio-economic disadvantage, housing precarity and demand for advocacy and support services.

The workshops formed part of the Roma PlaceAge project (2024-27), which aimed to explore experiences of healthy ageing among marginalised Roma populations. The approach was informed by principles of participatory and place-based research, with a focus on co-producing knowledge with policymakers, service providers and Roma advocates.

The workshops were designed to explore three interconnected research aims: (i) to examine organisational tensions, challenges and opportunities in embedding asset-based approaches to health and wellbeing for Roma communities; (ii) to capture community concerns, particularly those related to older Roma, as perceived by Roma community members themselves, advocacy organisations and service providers; and (iii) to identify policy and practice pathways for developing inclusive, place-based supports for healthy ageing within Roma communities.

### Process

The Knowledge Café is a dialogic, semi-structured method of group engagement that encourages reflective, open-ended discussion among participants (Brown & Isaacs, 2005). In each location, the café brought together 6–8 participants per table, with discussions organised around a ‘menu’ of questions addressing key themes of health access, housing, ageing-in-place and community support. Workshops included a mix of local authority officers, healthcare workers, community support staff, Roma advocates, Roma community members and civil society.

The café format was intentionally designed to foster equality, empowerment and informality in discussion. By drawing on a conversational rather than interrogative structure, the method helped to flatten hierarchies between professionals and community participants, creating space for more open and reciprocal exchange. Participants were encouraged to share personal reflections, practical insights and cultural knowledge without fear of judgment or bureaucratic scrutiny, enabling more inclusive dialogue across lines of institutional power and lived experience.

Each workshop lasted approximately three hours, beginning with introductory remarks and followed by three themed ‘courses’ of discussion: starter, main and dessert. Participants rotated tables between courses to maximise dialogue and cross-fertilisation of ideas, while facilitators remained to guide conversation and ensure continuity. Each group provided feedback to the wider audience after each ‘course’. Materials such as tablecloths, post-it notes and audio recording devices were used to capture both verbal and visual contributions.

### Sampling and Recruitment

A purposive sampling strategy was adopted to ensure the inclusion of professionals and community members with lived or practice-based experience of working with Roma communities. Participants were recruited through existing stakeholder networks and community partner organisations collaborating in the study.

In total, the workshops involved 61 participants across the three sites, including representatives from statutory organisations such as the National Health Service (NHS) and the police, employment and education providers, local government, advocacy groups such as Age UK and the Alzheimer’s Society, as well as voluntary and community sector organisations (see Table 1). While the primary voices captured were those of professionals and advocates, Roma perspectives were integrated both through direct participation (of community researchers recruited into the Roma PlaceAge project) and through the experiential knowledge of those working in close contact with Roma communities (a number of whom also self-identified as Roma). This hybrid format allowed for a nuanced understanding of both institutional and community perspectives.

**Table 1:** Participant characteristics by site

Site	Total Participants	Roma Community Members	Nationalities of Roma Participants	Professionals/ Practitioners	Example of Professional Roles
Govanhill (Glasgow)	26	8	Slovak, Czech and Romanian	18	NHS, ambulance services, community organisations, charities
Peterborough	14	8	Czech, Slovak and Romanian	6	NHS, local council, education providers, charities
Luton	21	2	Romanian	19	NHS, policing, local authority, voluntary sector, charities
Total	61	18	Czech, Slovak and Romanian	43	Mixed statutory and community roles

All quotes in the Findings section attributed to ‘Roma community member’ are direct contributions from Roma participants who attended the Knowledge Café workshops and shared their perspectives during discussions. Quotes attributed to specific professional roles (e.g. ‘healthcare practitioner,’ ‘voluntary and community sector organisation’) reflect perspectives shared by professionals and service providers.

### Data Management and Analysis

Following each workshop, all post-it notes and tablecloths were photographed and then transcribed. Audio recordings from each discussion table were transcribed verbatim. Data were then subject to reflexive thematic analysis, guided by the six-step approach of Braun and Clarke (2019, 2024). The analysis was conducted by a core team of three researchers with experience in qualitative methods and community-based research.

The process began with repeated reading of transcripts to ensure familiarisation. An initial coding framework was developed using a combination of descriptive (open) and interpretive (axial) coding (Boyatzis, 1998). Through iterative review and constant comparison, the research team identified cross-cutting themes and sub-themes. Coding discrepancies were discussed and refined in team meetings until consensus was reached. Reflexivity was maintained throughout, with researchers noting their positionality and potential influences on interpretation (Byrne, 2022). The diverse disciplinary and experiential backgrounds of the team including community-based practice, policy engagement and qualitative research informed an ongoing process of reflexive dialogue. Different perspectives were actively negotiated during the development and naming of themes, with particular attention paid to avoiding deficit-based framings and centring Roma agency and resilience. For instance, discussions about ‘mistrust’ of health services were reframed to acknowledge historical and ongoing discrimination rather than positioning Roma communities as inherently suspicious. Similarly, the theme of ‘home’ was shaped by insights from the Roma team member about the cultural and symbolic meanings of domestic space, family and belonging, which influenced how we interpreted participants’ accounts of ageing-in-place.

In addition to the core analytic team, the wider project team, comprising academics, NGO partners and Roma community members, also contributed to the interpretive process. Key themes were taken back to this broader group for discussion, refinement and validation. Many of these team members supported the facilitation of the Knowledge Café workshops and brought further lived experience of being Roma, enabling additional layers of cultural, linguistic and contextual insight to shape the final thematic structure. Their involvement provided an important collective check on interpretation, ensuring that emerging themes resonated with community understandings and practice-based experience.

The final themes were validated through cross-case comparison across the three sites and were grounded in both the voices of Roma individuals and those of practitioners and service providers. This allowed for a multi-layered exploration of the spatial, cultural and institutional dynamics shaping Roma experiences of ageing.

## Findings

The findings illuminate how older Roma individuals experience ageing in highly stratified spatial, institutional and cultural contexts. These themes presented highlight not only the structural exclusions faced by Roma communities but also their embedded strategies of resistance, adaptation and care that emerge in response. What unfolds is a complex portrait of ageing shaped by systemic marginalisation and the limitations of mainstream ageing and health and wellbeing frameworks to account for cultural difference.

### Exclusion in the Spaces of Health: The Disjuncture Between Roma Lives and Healthcare Systems

Practitioners across all three sites described healthcare as one of the most complex and exclusionary systems that Roma communities, particularly older people, must navigate. The barriers identified were not limited to eligibility or service access but were experienced spatially and culturally. Healthcare environments were often described as alienating, intimidating and misaligned with how Roma people live, communicate and seek support.

While language was consistently cited as a primary barrier, practitioners emphasised that the issue extended beyond the mere presence or absence of interpreters. Concerns were raised about the appropriateness of interpretation in terms of both dialect and cultural sensitivity and inherent prejudice and bias amongst some non-Roma interpreters:

“You have interpreters who come, but they don’t understand our words. They speak formal Romanian, not how we talk. So we nod, and they write something down, and the doctor says ‘OK’… but no one really knows what’s going on.” (Roma community member, Peterborough).

“The prejudices and the biases from the non-Roma interpreters quite often play a role in terms of how they are interpreting, but also what they are interpreting to the doctors or the professionals.” (Third sector organisation, Govanhill).

Service providers highlighted how written correspondence such as appointment letters, prescription notes and service notifications, were inaccessible to many older Roma individuals, particularly those with low literacy or those living in extended households where documents could easily go missing. These material and logistical obstacles were frequently misinterpreted by professionals as disinterest or non-compliance:

“We send letters and written communication to Roma patients regarding check-ups or operations but often on the assumption that they are seeing and able to understand those letters.” (Healthcare practitioner, Peterborough).

Digitalisation was another layer of exclusion. Practitioners noted how the shift to online platforms for booking appointments, registering with GPs, or accessing health information had effectively locked out many Roma older adults from primary care systems:

“A lot of health services now go towards the tools of being digital. What if you’re not digital? You don’t have the language.” (Health equalities practitioner, Luton).

Accounts of direct discrimination were also prevalent in workshop discussions. Providers relayed stories from service users who described being treated with suspicion, rushed through consultations, or denied dignity in their encounters with professionals. For many older Roma individuals, healthcare settings were a space of judgment and discomfort:

“They look at you in another way when they know you’re from the Roma community… And there’s this feeling that they don’t see you as a citizen or a person.” (Roma community member, Luton).

“Roma patients are treated differently, rushed through, not taken seriously, or accused of exaggerating their symptoms.” (Community engagement officer, Luton).

In response to these challenges, practitioners noted that many Roma individuals relied on informal support networks or opted to return to their country of origin for treatment. This was seen not as a rejection of the UK health system per se, but as a rational response to accumulated experiences of exclusion and ‘feeling lost’ within health and care systems:

“In Romania, I know how it works. Here, I always feel lost. Like I’m not meant to be there.” (Roma community member, Peterborough).

In relation to gendered dynamics of care support, the ‘invisible labour’ of older women was frequently discussed. Despite their own declining health, many continued to take on significant caregiving roles. This involved not only caring for grandchildren and managing domestic responsibilities, but also supporting older relatives, including spouses and siblings, with health needs and daily living tasks. Yet, this extensive caregiving role was rarely acknowledged within service encounters or formal assessments, rendering their contributions invisible and their own needs overlooked:

“Older women often do the most work in the family… caring, cooking… yet they’re not seen as carers by services.” (Third sector organisation, Luton).

At the same time, practitioners also reflected on how gendered expectations around masculinity shaped older Roma men’s healthcare engagement. Several participants noted that men were often less likely to seek help proactively, viewing medical intervention as a last resort. This reluctance was attributed to cultural ideals of toughness, fear of appearing weak, or distrust rooted in earlier experiences of exclusion or discrimination:

“A lot of the older men won’t go to the doctor until they absolutely have to. It’s like, ‘I’ll deal with it myself.’ Sometimes that means conditions get worse before they even get looked at.” (Frontline health worker, Govanhill).

Overall, this theme underscores how healthcare exclusion is not only a logistical issue, but a deeply spatial and relational one. The physical and symbolic architectures of healthcare, its languages, technologies, layouts and institutional cultures, fail to align with the realities of Roma life. In the accounts shared by service providers, older Roma individuals are not refusing care, but rather navigating a system in which they feel both alien and invisible:

“We need to stop asking why they’re not engaging and start asking what it would look like to create a space they can trust.” (Education provider, Govanhill).

Rather than viewing healthcare disengagement as reluctance or resistance, a deficit model long applied to Roma populations experiencing disproportionately high morbidity and premature mortality (Sarafian et al., 2024; Orton et al., 2019; Földes & Covaci, 2012), this theme highlights how exclusion is produced through the interplay of language barriers, limited digital and written literacy, gendered care roles and experiences of stigma and indignity. Existing research, both in the UK (Gaiser, 2017; Ali et al., 2024) and internationally (Parekh & Rose, 2011; Plavnicka et al., 2025), has similarly shown how health systems are structured around implicit expectations of cultural and linguistic capital, rendering routine processes inaccessible to many Roma individuals. However, very little work has examined how these barriers compound in later life, or how age, gender, migration history and literacy intersect to deepen marginalisation.

The findings here therefore underscore that the challenge is not simply *what* services are offered, but *how* they are designed and for whom. Creating spaces where older Roma feel recognised, safe and respected requires attention to the relational and structural dimensions of exclusion, and to models of care that better align with the lived realities, histories and cultural values of Roma communities.

### The Fragility of Ageing-in-Place: Housing Precarity and the Limits of Autonomy

While practitioners recognised that many older Roma individuals wished to remain in their own homes and communities as they aged, this ideal of ageing-in-place was often described as fragile, constrained by housing precarity, insecurity and exclusion from mainstream support systems. Ageing at home, although symbolically and emotionally significant in Roma culture, was frequently dependent on informal arrangements and occurred in conditions that undermined safety, health and dignity.

Across all three Knowledge Café sites, housing emerged as a critical barrier to healthy ageing. Practitioners reported that older Roma individuals commonly lived in overcrowded, damp, or poorly maintained private rental housing, often without clear tenancy rights or poor recourse to landlords for repairs and with even less knowledge of their legal rights. These physical environments were seen to exacerbate existing health conditions such as arthritis, respiratory illnesses and mobility challenges and to contribute to a sense of instability and fear:

“The conditions the Roma community live in… Mould, broken windows, no heating in the winter. How can you age well in those conditions?” (Housing support worker, Luton).

“We go to homes where older people are living with young children, multiple families sharing two rooms. That creates its own challenges for ageing.” (Healthcare worker, Peterborough).

There was a clear sense from support workers and practitioners that while older Roma individuals expressed a strong desire to remain in their homes, they were rarely presented with viable alternatives. Mainstream housing support was often inaccessible and many participants spoke of the frustrations of supporting clients through social housing application processes that were slow, bureaucratic and discriminatory:

“They make us wait years for council housing, but others get it faster. Why?” (Roma community member, Govanhill).

“We had one older woman who needed an adapted property because of her mobility. Getting aids and adaptations is problematic.” (Voluntary and community sector organisation, Peterborough).

Support workers consistently framed housing insecurity not as a standalone issue, but as a barrier that intersected with health, access to care and social inclusion. Poor housing conditions were said to discourage engagement with services either because people were ashamed of their living situation or because they feared that inviting professionals into their homes would trigger unwanted scrutiny or involvement from other agencies:

“They won’t let anyone in because they think they’ll be judged, or worse, evicted. That fear is real.” (Community advocate, Luton).

These concerns were particularly acute for older women and for those living in extended family settings, where care responsibilities and privacy were often entangled. While practitioners acknowledged the strength of Roma intergenerational family structures, they also recognised that these arrangements could place pressure on older individuals, who were expected to cope with less than optimal housing circumstances or with expectations of providing childcare so that younger family members could work:

“There’s this romantic idea that Roma families look after their elders. Sometimes they do. But sometimes older people are just expected to cope.” (Healthcare provider, Peterborough).

The symbolic importance of home, however, was never in question. Practitioners reported that institutional care was rarely viewed as acceptable and that the very idea of a care home was associated with abandonment or loss of cultural identity. For many, remaining in their home, no matter how inadequate, was seen as essential to maintaining autonomy and familial bonds:

“Roma people don’t go to old people’s homes. That is not our way.” (Roma community member, Luton).

Yet practitioners also expressed concern that while many older Roma individuals do wish to remain in their homes and communities, this aspiration is often constrained by substandard housing and lack of accessible support. The desire to age in place was clear, but it was often not matched by the conditions needed to do so safely and with dignity, such as adequate space, repairs, adaptations or proximity to trusted services:

“We need to move beyond saying ‘they want to stay at home.’ We have to ask what kind of home? What kind of support? It’s not just about preference. It’s about rights and access.” (Local authority officer, Govanhill).

Whilst poor-quality housing is a challenge shared by many migrant and low-income communities (Kristiansen et al., 2016), the experiences of older Roma adults reflect a distinctive layering of vulnerabilities. Barriers to engaging with housing authorities and support services are intensified by pre-migration histories of discrimination, limited awareness of legal entitlements, and deep-seated fears of institutional scrutiny, factors well documented in Roma health and social exclusion research (Kostka et al., 2024; Valero et al., 2021). These intersecting dynamics constrain opportunities to secure safe, adequate housing and to access the adaptations or support required for ageing-in-place.

Taken together, this theme highlights the tension between the strong cultural preference to age within the home and family environment, and the structural barriers that render this aspiration precarious. While intergenerational support remains a vital resource, it is often insufficient to compensate for systemic failures in housing policy, landlord regulation and accessible adaptation services. Without targeted intervention, the very spaces that should provide stability, dignity and belonging in later life risk becoming sources of vulnerability, reinforcing isolation or, at worst, exposing older Roma adults to harmful levels of insecurity and unwanted institutional involvement.

### Isolation in the Everyday: Social Disconnection and Place Belonging

Practitioners across all sites described a growing sense of social disconnection affecting older Roma individuals, particularly women and those with declining mobility or chronic health conditions. While social isolation is widely recognised as a public health concern in ageing populations, practitioners framed the isolation of older Roma people as distinct: shaped not only by individual health or mobility status, but by spatial marginalisation, cultural exclusion and the erosion of Roma-specific public and community spaces.

A key factor repeatedly raised was the gendered dynamic of social life. While Roma men were described as more likely to continue informal social networks in public places, older women were said to experience a steady contraction of their social world:

“Men might go out, meet friends, but for many women, social life shrinks as they age. They stay in the home, they don’t go to English groups, and they’re not in touch with what’s available.” (Health mediator, Luton).

This contraction was compounded by the disappearance of informal gathering places where older Roma individuals previously felt at ease. Practitioners spoke of community centres that had closed, spaces that had been gentrified and public places that had become sites of surveillance rather than welcome during the period since they had moved to the UK. Thus, there was a weakened sense of place ownership and reduced opportunities for culturally welcoming (temporary) appropriation of public space:

“We used to meet outside, but now there are no places left for us. Every space we had has been taken over.” (Roma community member, Peterborough).

In Govanhill, some of the practitioners described the spatial tension of regeneration. While public investments were being made in green spaces, pedestrian routes and park infrastructure, these spaces were not always experienced as inclusive or safe by older Roma. Without targeted design, language support, or community presence, even well-meaning initiatives could reinforce the feeling of not belonging:

“The spaces exist but they’re not ‘for us.’ They don’t feel like ours. That’s why we stick with our homes and meeting up with families.” (Roma community member, Govanhill).

Mobility emerged as a key intersecting factor, with practitioners describing how basic urban features such as benches, crossings and clear signage, could make the difference between being part of a neighbourhood and being functionally confined to the home. Older people with mobility or visual impairment, for example, were less likely to venture out in neighbourhoods that lacked places to rest or where public transport was infrequent and difficult to navigate:

“There are no benches to sit down when I walk to the shop. If I get tired, I have to keep going or sit on the ground.” (Roma community member, Peterborough).

While mainstream community groups and opportunities for formal participation do exist in all three sites, practitioners emphasised that these were often culturally misaligned and underutilised by older Roma. Language resurfaced again as a barrier, but so too were unspoken norms around hospitality, participation and formality:

“The day centre is open to everyone in theory, but in practice, we don’t see Roma people there. They don’t feel it’s for them. It’s English-only, it’s structured and it doesn’t have their food or their music.” (Health inequalities practitioner, Luton).

Some participants highlighted the loss of culturally specific community-led events including Roma dances, religious celebrations and informal food-sharing spaces that traditionally served to bring members of the community together. Austerity, funding competition and policy shifts towards generic “diversity and inclusion” programming were seen to have stripped away Roma-specific cultural anchors:

“There used to be a space where older women cooked, brought their kids, spoke their language. That’s gone. Now they’re told to go to ‘multicultural’ events where they don’t know anyone and nothing is familiar.” (Social support worker, Govanhill).

Several practitioners noted the psychological toll this isolation takes, not only in terms of loneliness, but in the erosion of cultural identity, agency and visibility in later life. While some older Roma continued to play key roles in family life including caring for grandchildren and passing down traditions, others were described as withdrawing, becoming “invisible” in their communities and to services:

“There’s a whole group of older Roma who have just disappeared from public life. They’re in the background… but not connected, not represented, not supported.” (Education provider, Peterborough).

Yet, practitioners also reflected on the untapped strengths of these same older people including their knowledge, leadership and resilience and emphasised the need to reframe ageing as not just decline or dependency, but as a time when contributions can still be made, if spaces for expression and recognition are restored:

Older Roma have so much to give… stories, advice, support… but they need spaces where they feel safe and respected. Right now, those spaces are not there.

(Voluntary and community sector organisation, Luton)

In summary, what practitioners described was not just a lack of social contact but a profound sense of spatial and cultural disconnection in old age. Isolation was both a symptom and a consequence of systems and spaces that fail to accommodate Roma ways of being, gathering and ageing. While these experiences resonate with broader research on ageing among migrant communities, such as the lifecourse place-based disadvantage documented among older South Asian communities (Cotterell et al., 2025) and the social exclusion experienced by older migrant groups more generally (Joshi et al., 2025), older Roma adults occupy a particularly liminal post-migration position. Roma communities in the UK are often relatively small, politically marginal and characterised by limited bridging capital, reducing opportunities to influence local decision-making or access mainstream social infrastructures. As the first generation ageing in place following recent migration, many older Roma individuals cannot draw on longstanding community institutions or accumulated social capital and they continue to carry the weight of pre-migration discrimination and exclusion, further inhibiting their capacity to assert needs or seek support from statutory services.

Without renewed investment in culturally rooted community infrastructure, meaningful co-production and the creation of accessible places where older Roma can gather, participate and feel a sense of ownership, their social exclusion is likely to deepen further.

### Beyond the Margins: Community Innovation and Place-Based Change

Amid widespread accounts of systemic exclusion, housing precarity and cultural marginalisation, the Knowledge Café workshops also revealed a persistent sense of possibility among practitioners and community leaders. Across all three sites, participants identified ways in which Roma-led or Roma-facing initiatives could offer not only practical assistance but also social connection, dignity and culturally resonant forms of support.

The role of Roma health mediators was raised across all workshops, individuals from within the community trained to act as a bridge between Roma families and formal health services. Participants highlighted how the credibility, cultural competence and language skills of health mediators could help overcome many of the barriers associated with accessing supports in old age. Mediators were not just seen as interpreters but cultural brokers, educators and advocates who could ‘translate’ the logic of systems while simultaneously protecting the dignity and preferences of older Roma:

“Health mediators could make all the difference. People to trust because they speak the Roma language and understand their lives.” (Public health practitioner, Luton).

However, practitioners noted that in the UK, where the health mediator model is far less established than in Central and Eastern Europe (Council of Europe, 2016; WHO, 2019), roles are often limited to short-term projects or narrow service areas such as maternity or early years. As one participant observed:

“We’ve seen how well this works for different groups. But then the funding ends, or the person moves on, and the whole connection disappears.” (Voluntary and community sector organisation, Peterborough).

The European Roma Grassroots Organisation (ERGO, 2022) similarly highlights the value of health navigators across the life-course, noting that older Roma people face particular challenges linked to digital exclusion, increased morbidity, shorter life expectancy, poverty, discrimination and the lack of tailored services. These barriers were strongly echoed in our findings.

Alongside the need for health navigators, participants also emphasised the vital role of community-based spaces in supporting older people. They described community drop-in centres, Roma-led advice hubs and grassroots initiatives as key spaces for engagement. In these environments where ‘tea and conversation’ preceded the necessity of dealing with bureaucracy or support with completing forms, where face-to-face contact was prioritised, and where staff understood the realities of Roma life, older individuals were more likely to ask for help, access services or participate in programmes:

“People won’t go to the council offices, but they’ll come here [to the Roma centre] for help. It’s a space where they feel welcome, not judged.” (Voluntary and community sector organisation, Govanhill).

Yet these community spaces too faced precarity. Competition for funding, bureaucratic reporting requirements and the shift towards ‘digital-first’ commissioning models were said to threaten the relational ethos at the heart of effective Roma engagement:

“We’re constantly justifying our existence… writing funding bids instead of building relationships. And the big organisations win because they know the funding language.” (Voluntary and community sector organisation, Luton).

Despite these constraints, practitioners spoke with clarity and creativity about what culturally competent, place-based ageing support could look like. Ideas included mobile outreach teams that could bring health advice directly to homes; multi-service hubs co-located in trusted community spaces; older adult peer programmes; and intergenerational projects grounded in Roma cultural values:

“You need to meet people where they are… not just physically, but emotionally and culturally. That means starting with the community and building out, not dropping in from the top.” (Healthcare provider, Peterborough).

Some reflected on the importance of not only tailoring services to Roma needs, but involving Roma communities in the design, delivery and governance of those services. Co-production was framed not simply as a buzzword but as a moral and practical necessity:

“It’s not just about being listened to… it’s about being part of the solution. That’s what gives people a reason to come, to stay, to lead.” (Roma community leader, Luton).

This vision of co-production, however, was tempered by realism. Participants noted that the capacity to engage meaningfully was not always evenly distributed. Older adults, especially those with limited English or poor digital access, often needed facilitation or accompaniment, for example, being supported by a trusted community advocate, health mediator or family member, to fully participate in discussions around service design. Nevertheless, the desire to centre older Roma voices was strong:

“We hear from the loudest voices. But older Roma have wisdom too. They’ve lived through everything. We need to find ways for their voices to shape what we do.” (Local authority officer, Govanhill).

Overall, this theme revealed a dual reality. While systems remained exclusionary, practitioners and community advocates were actively experimenting with ways to meld those systems toward equity and inclusion. These innovations were deeply place-based, not just in location, but in ethos. They began with what was already present in Roma communities: resilience, cultural richness and mutual care. But to move beyond isolated success stories, participants argued, these culturally congruent models must be recognised, resourced and embedded at a structural level.

In summary, the findings point to a significant disconnect between the top-down, system-oriented logic of most ageing policy and the relational, place-based realities of ageing in Roma communities. Addressing this requires more than adaptation. It requires a fundamental rethinking of what it means to age well in the context of membership of a marginalised community.

## Discussion: Rethinking Healthy Ageing Through the Lens of Roma Lives and Places

The findings reveal a profound disjuncture between dominant models of healthy ageing and the lived realities of older Roma individuals. While the global policy landscape increasingly embraces concepts such as ‘ageing-in-place’ and age-friendly community frameworks (Greenfield & Buffel, 2022; WHO, 2007), these approaches often rest on assumptions that overlook the lived experiences of structurally marginalised populations. For Roma communities, ageing is not just shaped by the ageing body or time, but by layered systems of exclusion - material, spatial, institutional, and epistemic - that constrain access to the very supports these frameworks presume to be universal.

Existing research has highlighted that ageing-in-place, although framed as a progressive goal, often pays insufficient attention to the unevenness of place itself (Peace et al., 2007; Wiles et al., 2012; Yarker et al., 2024). For the older Roma described in this study, home may be a site of cultural belonging and familial attachment but remaining there can become a source of risk when that home is overcrowded, insecure or stigmatised. This aligns with previous critiques that highlight the risks of romanticising ageing-in-place without attending to the quality of housing, community relations or structural support (Sixsmith et al., 2014; Woolrych et al., 2021). As our findings show, for many Roma individuals, ‘ageing-in-place’ is less a proactive choice than a constrained necessity, shaped by discrimination in the housing market, poor access to adaptations and a broader policy infrastructure that fails to account for their cultural and spatial realities.

We also contribute to the literature on the spatialised nature of healthcare exclusion (Janevic et al., 2011; McFadden et al., 2018). Services remain structured in ways that implicitly exclude Roma people: digital-only access, language barriers, rigid systems and institutional cultures that foster mistrust and stigma. These exclusions are cumulative, undermining not only physical health but also older Roma individuals’ sense of visibility, legitimacy and belonging. As in other racialised or migrant groups (Quigley et al., 2022; Zubair & Norris, 2015), disengagement from formal systems is not apathy but a rational response to systemic disregard, cultural misrecognition and prior trauma.

Gender emerged as a key axis of exclusion and resilience. Our findings extend understandings of gendered ageing by showing how older Roma women, despite their own health challenges, are relied upon for extensive informal care, supporting grandchildren, adult children and even older male relatives. These caregiving roles are rarely recognised by services, contributing to a form of double invisibility (Földes & Covaci, 2012; Plaza del Pino et al., 2022). The absence of culturally safe and gender-sensitive third spaces intensifies their social isolation, echoing findings from broader intersectional ageing research (Carr et al., 2015; Yazdanpanahi & Woolrych, 2023).

Taken together, the findings underscore three core dynamics: (1) structural exclusions embedded in health and housing systems; (2) the gendered and cultural dimensions of care and resilience; and (3) the emergence of place-based, community-led responses that reframe what ageing-in-place means for marginalised groups.

Crucially, our research challenges deficit-based framings of Roma communities and other marginalised groups as passive recipients of care. Instead, we highlight everyday forms of resilience and cultural strength. Roma health mediators, bilingual advocates and grassroots drop-in centres exemplify what Cosco et al. (2017) discuss as resilience enabled through individual, social and environmental resources, a form of support that enables older adults to age with dignity through networks of mutual aid, cultural familiarity and local embeddedness. This perspective contributes to emerging understandings of resilience in old age as socially and culturally situated, rather than individualised (Bailey et al., 2018; Cutchin, 2017). We argue that interdependence should be recognised as central to ageing in place policy, not only as a cultural value but as a structural necessity in later life, particularly for those living in contexts of inequality. Our findings support calls for a shift toward relational and collective models of ageing (Fine & Glendinning, 2005; Phillipson, 2020), where reciprocity, trust and shared knowledge become the foundations of inclusive support.

A key contribution of this study is its attention to the local and relational dimensions of care, highlighting how place-based infrastructures need to be shaped in collaboration with Roma communities. Community-led initiatives were viewed as most effective when they were grounded in local trust, cultural knowledge and embedded networks. These actors, including Roma health mediators, grassroots advocates and community-based practitioners, represent alternative models of care grounded in relational ethics and situated expertise. The community hubs they work through can act as sites of service delivery, but also spaces of ownership, belonging and informal accountability. Together, these actors and settings challenge the extractive, standardised logics of mainstream provision, offering instead a vision of care as a social and spatial practice rooted in respect, reciprocity and lived experience (Hayes et al., 2025; Miranda et al., 2019).

However, these innovations remain fragile. Our participants described a policy landscape in which Roma community organisations are expected to deliver significant outcomes with minimal support, short-term funding and little voice in policymaking. This reflects the wider neoliberal drift in ageing policy, where responsibility for adaptation is shifted to communities without structural investment (MacNicol, 2015; Phillipson, 2020). The framing of Roma as ‘hard to reach’ obscures the more pressing reality of ‘hard-to-access’ systems that routinely exclude minoritised communities across healthcare, housing and social support landscapes.

Our findings therefore have broader relevance beyond Roma communities, with clear implications for how we design, fund and deliver services for all marginalised older adults. While the nature and histories of exclusion differ, many groups including migrant communities, older LGBTQ + individuals and racially minoritised populations, encounter parallel forms of marginalisation, such as structurally inaccessible services, culturally unsafe environments and limited recognition of diverse ways of ageing (Buffel, 2017; Kneale et al., 2021). If healthy ageing is to be truly inclusive, it must be built *with*, not *for*, communities. This requires genuine collaboration, long-term relationships and recognition of lived expertise as valid knowledge. Moreover, participatory approaches must be resourced in ways that account for language, literacy, digital exclusion and intergenerational dynamics, conditions especially relevant in but not exclusive to Roma contexts.

Ultimately, this study reinforces the need to reframe healthy ageing not as an abstract, universalised goal, but as a situated, relational and political process shaped by the material and symbolic geographies of everyday life. For older Roma, exclusion from healthcare, housing and social infrastructure is not incidental but spatially patterned, rooted in a lack of culturally safe places to age, gather or access support. This reflects what has been termed spatial injustice (Purcell, 2002; Soja, 2010) or the systematic denial of the right to participate, belong and thrive within shared urban environments (Menezes et al., 2023). A more inclusive gerontology must take seriously the questions of place justice, recognising the importance of dignity, cultural identity and spatial belonging as foundations for equitable ageing, across all communities experiencing marginalisation.

## Conclusion

This study has demonstrated that for Roma communities, healthy ageing is shaped less by individual behaviours than by the spatial, institutional and cultural conditions in which ageing unfolds. Older Roma face layered barriers to healthcare, secure housing and social participation, yet also display remarkable resilience and resourcefulness. Place-based, co-produced and culturally embedded interventions hold significant promise for addressing these disparities but only if ageing policy frameworks are reimagined through the lens of equity, justice and relational care. Future work must move beyond inclusion into transformation to centring Roma voices in the co-design of environments and systems where ageing can be not only possible, but meaningful.

The Knowledge Café workshops presented in this paper provided a valuable platform for inclusive dialogue, enabling Roma participants, practitioners and service providers to come together in non-hierarchical, conversational spaces. These settings fostered mutual learning, and the sharing of diverse perspectives on ageing, care and place. A key strength of this format was its ability to surface both common concerns and points of disagreement across generations, roles and cultural backgrounds, which participants viewed as essential to challenging assumptions and enriching understanding. The workshops also helped build relationships between the various participant groups, offering a foundation for future collaboration.

To deepen understanding of ageing within Roma communities, future research would benefit from more experiential and in-depth approaches that engage directly with older Roma individuals. Mobile methods, storytelling and participatory approaches could offer richer insight into the spatial and emotional dimensions of later life and help surface the everyday realities of older Roma ageing-in-place. Secondly, there is a need for comparative work across European contexts examining how different welfare regimes, housing systems and integration policies shape possibilities for healthy ageing among Roma populations. Finally, given the fragmented and under-resourced nature of community-led provision, there is an urgent need to establish, adequately resource and evaluate models such as health mediation, Roma community hubs and co-designed service pathways, to build an evidence base that can inform long-term policy investment. 

## Data Availability

The data that support the findings of this study are not openly available due to reasons of sensitivity and are available from the corresponding author upon reasonable request.

## References

[CR1] Alexiadou, E. A. (2023). Identifying a Human Rights Approach to Roma Health Vulnerabilities and Inequalities in Europe: From Concept to Action. *Human Rights Review*, *24*(3), 413–431. 10.1007/s12142-023-00684-6 10.1007/s12142-023-00684-6 PMC10144874 37362822

[CR2] Ali, N., Mackay, F., & Randhawa, G. (2024). *Talk, Listen, Change (TLC): Engaging in Dialogue with the Luton Roma Community on Access to Healthcare Services*. University of Bedfordshire: Institute for Health Research. 10.3389/fpubh.2026.1716479 PMC13071022 41982890

[CR3] Bailey, C., Gilroy, R., Reynolds, J., Douglas, B., Saaremets, C. W., Nicholls, M., Warwick, L., & Gollan, M. (2018). Ageing in place: Creativity and resilience in neighbourhoods. In A. Goulding, B. Davenport, & A. Newman (Eds.), *Resilience and ageing: Creativity, culture and community* (pp. 157–180). Policy. 10.1332/policypress/9781447340911.003.0008

[CR4] Boyatzis, R. E. (1998). *Transforming qualitative information: Thematic analysis and code development*. Sage.

[CR5] Braun, V., & Clarke, V. (2019). Reflecting on reflexive thematic analysis. *Qualitative Research in Sport Exercise and Health*, *11*(4), 589–597. 10.1080/2159676X.2019.1628806

[CR6] Braun, V., & Clarke, V. (2024). Supporting best practice in reflexive thematic analysis reporting in Palliative Medicine: A review of published research and introduction to the Reflexive Thematic Analysis Reporting Guidelines (RTARG). *Palliative medicine*, *38*(6), 608–616. 10.1177/02692163241234800 38469804 10.1177/02692163241234800 PMC11157981

[CR7] Brown, J., & Isaacs, D. (2005). *The World Café: Shaping our futures through conversations that matter*. Berrett-Koehler.

[CR8] Bueno, A. M. (2023). Intercultural Mediation for the Roma Communities: The Belgian Healthcare Case. *Treballs de la Societat Catalana de Geografia*, *94*, 59–81. 10.2436/20.3002.01.227

[CR9] Buffel, T. (2017). Ageing migrants and the creation of home: Mobility and the maintenance of transnational ties. *Population Space and Place*, *23*(5), e1994. 10.1002/psp.1994

[CR10] Buffel, T., & Phillipson, C. (2024). *Ageing in place in urban environments: Critical perspectives*. Taylor & Francis.

[CR11] Byrne, D. (2022). A worked example of Braun and Clarke’s approach to reflexive thematic analysis. *Quality & Quantity*, *56*(3), 1391–1412. 10.1007/s11135-021-01182-y

[CR12] Carr, A., Biggs, S., & Kimberley, H. (2015). Ageing, Diversity and the Meanings of Later Life: Cultural, Social and Historical Models to Age by. *Contemporary Readings in Law and Social Justice*, *7*(1), 7–60.

[CR13] Cook, B., Wayne, G. F., Valentine, A., Lessios, A., & Yeh, E. (2013). Revisiting the evidence on health and health care disparities among the Roma: a systematic review 2003–2012. *International Journal of Public Health*, *58*, 885–911. 10.1007/s00038-013-0518-6 24096986 10.1007/s00038-013-0518-6

[CR14] Cosco, T. D., Howse, K., & Brayne, C. (2017). Healthy ageing, resilience and wellbeing. *Epidemiology and Psychiatric Sciences*, *26*(6), 579–583. 10.1017/S2045796017000324 28679453 10.1017/S2045796017000324 PMC6998987

[CR15] Cotterell, N., Buffel, T., Nazroo, J., & Qualter, P. (2025). Loneliness among older ethnic minority people: exploring the role of structural disadvantage and place using a co-research methodology. *Ethnic and Racial Studies*, *48*(1), 206–228. 10.1080/01419870.2024.2311833

[CR16] Council of Europe (2016). *ROMED: Intercultural mediation for Roma inclusion*. Retrieved December 1, 2025 from https://www.coe.int/en/web/romed

[CR17] Cutchin, M. P. (2017). Active relationships of ageing people and places. In M. Skinner, G. Andrews, M. Cutchin (Eds.), *Geographical Gerontology* (pp. 216–228). Routledge. 10.4324/9781315281216-17

[CR18] EPHA (2020). *Health Inequalities: A Persistent Obstacle for Roma Equality and Inclusion.* European Public Health Alliance. Retrieved June 3, 2025, from https://epha.org/health-inequalities-a-persistent-obstacle-for-roma-equality-and-inclusion/

[CR19] ERGO (2022). *Roma access to adequate healthcare and long-term care*. Retrieved December 1, 2025 from Ergo-2022-access-healthcareWEB.pdf.

[CR20] European Disability Forum. (2022). *Briefing on discrimination and social exclusion of Roma with disabilities*. Retrieved June 3, 2025, from https://www.edf-feph.org/publications/briefing-on-discrimination-and-social-exclusion-of-roma-with-disabilities/

[CR21] European Commission. (2020). *Roma equality, inclusion and participation in the EU*. Retrieved December 1, 2025 from https://commission.europa.eu/strategy-and-policy/policies/justice-and-fundamental-rights/combatting-discrimination/roma-eu/roma-equality-inclusion-and-participation-eu_en

[CR22] Fine, M., & Glendinning, C. (2005). Dependence, independence or inter-dependence? Revisiting the concepts of ‘care’ and ‘dependency’. *Ageing & Society*, *25*(4), 601–621. 10.1017/S0144686X05003600

[CR23] Földes, M. E., & Covaci, A. (2012). Research on Roma health and access to healthcare: state of the art and future challenges. *International Journal of Public Health*, *57*(1), 37–39. 10.1007/s00038-011-0312-2 21972058 10.1007/s00038-011-0312-2 PMC3282005

[CR24] FRA (2022). *Roma in 10 European Countries-Main Results.* European Union Agency for Fundamental Rights. Retrieved June 3, 2025, from https://fra.europa.eu/sites/default/files/fra_uploads/fra-2022-roma-survey-2021-main-results_en.pdf

[CR25] Gaiser, L. (2017). Language barriers and access to health care among Roma migrants: A case study of Manchester, UK. *MigRom*. Retrieved 1 December 2025 from http://migrom.humanities.manchester.ac.uk/wp-content/uploads/2017/11/Roma-Access-to-Health-Care-Leonie-Gaiser.pdf

[CR26] Greenfield, E. A., & Buffel, T. (2022). Age-friendly cities and communities: Research to strengthen policy and practice. *Journal of Aging & Social Policy*, *34*(2), 161–174. 10.1080/08959420.2022.2049573 35311484 10.1080/08959420.2022.2049573

[CR27] Greenfields, M. (2017). Good practice in working with Gypsy, Traveller and Roma communities. *Primary Health Care*, *27*(10), 24–29. 10.7748/phc.2017.e1263

[CR28] Greenfields, M., & Ryder, A. (2012). Research with and for Gypsies, Roma and Travellers: Combining policy, practice and community in action research. In J. Richardson, & M. Ryder (Eds.), *Gypsies and Travellers* (pp. 151–168). Policy.

[CR29] Hayes, K., Coxon, K., & Bye, R. A. (2025). Rural and remote health care: the case for spatial justice. *Rural and remote health*, *25*(1), 1–9. 10.22605/RRH8580 10.22605/RRH8580 39869933

[CR30] Janevic, T., Sripad, P., Bradley, E., & Dimitrievska, V. (2011). There’s no kind of respect here A qualitative study of racism and access to maternal health care among Romani women in the Balkans. *International Journal for Equity in Health*, *10*, 1–12. 10.1186/1475-9276-10-53 22094115 10.1186/1475-9276-10-53 PMC3226439

[CR31] Joshi, M., Finney, N., & Hale, J. M. (2025). Loneliness and social isolation of ethnic minority/immigrant older adults: a scoping review. *Ageing and Society*, *45*(7), 1395–1425. 10.1017/S0144686X24000205

[CR32] Kneale, D., Henley, J., Thomas, J., & French, R. (2021). Inequalities in older LGBT people’s health and care needs in the United Kingdom: a systematic scoping review. *Ageing & Society*, *41*(3), 493–515. 10.1017/S0144686X19001326 34531622 10.1017/S0144686X19001326 PMC8423450

[CR33] Kostka, J., Greenfields, M., Felja, D., Boyce, M., Radley, C., & Coker, S. (2024). Navigating the labyrinth: Exploring the experiences of Roma families with child protection services. *Critical and Radical Social Work*, 1–18. 10.1332/20498608Y2024D000000065

[CR34] Kristiansen, M., Razum, O., Tezcan-Güntekin, H., & Krasnik, A. (2016). Aging and health among migrants in a European perspective. *Public Health Reviews*, *37*(1), 20. 29450062 10.1186/s40985-016-0036-1 PMC5809957

[CR35] MacNicol, J. (2015). *Neoliberalising old age*. Cambridge University Press.

[CR36] Málovics, G., Creţan, R., Méreiné Berki, B., & Tóth, J. (2019). Urban Roma, segregation and place attachment in Szeged. *Hungary Area*, *51*(1), 72–83.

[CR37] McFadden, A., Siebelt, L., Gavine, A., Atkin, K., Bell, K., Innes, N., & MacGillivray, S. (2018). Gypsy, Roma and Traveller access to and engagement with health services: a systematic review. *The European Journal of Public Health*, *28*(1), 74–81. 10.1093/eurpub/ckx226 29346666 10.1093/eurpub/ckx226

[CR38] Menassa, M., Stronks, K., Khatami, F., Díaz, Z. M. R., Espinola, O. P., Gamba, M., & Franco, O. H. (2023). Concepts and definitions of healthy ageing: a systematic review and synthesis of theoretical models. *EClinicalMedicine*, *56*. 10.1016/j.eclinm.2022.101821 10.1016/j.eclinm.2022.101821 PMC9852292 36684393

[CR39] Menezes, D., Woolrych, R., Sixsmith, J., Makita, M., Smith, H., Fisher, J., & Murray, M. (2023). You really do become invisible’: examining older adults’ right to the city in the United Kingdom. *Ageing & Society*, *43*(11), 2477–2496.

[CR40] Miranda, D. E., Garcia-Ramirez, M., Balcazar, F. E., & Suarez-Balcazar, Y. (2019). A community-based participatory action research for Roma health justice in a deprived district in Spain. *International Journal of Environmental Research and Public Health*, *16*(19), 3722. 10.3390/ijerph16193722 31581695 10.3390/ijerph16193722 PMC6801624

[CR41] Mózes, N., Takács, J., Ungvari, Z., & Feith, H. J. (2024). Assessing disparities in health and living conditions: a comparative study of Hungarian-speaking Roma and non-Roma women across Hungary, Romania, and Slovakia. *Frontiers in Public Health*, *12*, 1438018. 10.3389/fpubh.2024.1438018 39234083 10.3389/fpubh.2024.1438018 PMC11371694

[CR42] Orton, L., de Anderson, R., Stojanovski, K., Gamella, J. F., Greenfields, M., La Parra, D., & Whitehead, M. (2019). Roma populations and health inequalities: a new perspective. *International Journal of Human Rights in Healthcare*, *12*(5), 319–327. 10.1108/IJHRH-01-2019-0004 32082612 10.1108/IJHRH-01-2019-0004 PMC7032950

[CR43] Pani-Harreman, K. E., Bours, G. J., Zander, I., Kempen, G. I., & van Duren, J. M. (2021). Definitions, key themes and aspects of ‘ageing in place’: a scoping review. *Ageing & Society*, *41*(9), 2026–2059. 10.1017/S0144686X20000094

[CR44] Parekh, N., & Rose, T. (2011). Health inequalities of the Roma in Europe: A literature review. *Central European Journal of Public Health*, *19*(3), 139–142. 10.21101/cejph.a3661. 22026288 10.21101/cejph.a3661

[CR45] Peace, S., Holland, C., & Kellaher, L. (2007). *Environment and identity in later life*. McGraw-Hill Education.

[CR46] Phillipson, C. (2020). Austerity and precarity: individual and collective agency in later life. In A. Grenier, C. Phillipson, & R. Settersten (Eds.), *Precarity and Ageing* (pp. 215–236). Policy.

[CR47] Plavnicka, J. M., Veselska, Z. D., & Bobakova, D. F. (2025). Barriers to primary health care: perspectives of marginalized Roma women and healthcare professionals. *BMC Health Services Research*, *25*(1), 1284. 10.1186/s12913-025-13482-2 41039520 10.1186/s12913-025-13482-2 PMC12492820

[CR48] Plaza del Pino, F. J., Arrogante, O., Gallego-Gómez, J. I., Simonelli-Muñoz, A. J., Castro-Luna, G., & Jiménez-Rodríguez, D. (2022). Romani women and health: The need for a cultural-safety based approach. *Healthcare, 10*(2), 271. 10.3390/healthcare10020271 10.3390/healthcare10020271 PMC8872497 35206885

[CR49] Purcell, M. (2002). Excavating Lefebvre: The right to the city and its urban politics of the inhabitant. *Geojournal*, *58*(2–3), 99–108. 10.1023/B:GEJO.0000010829.62237.8f

[CR50] Quigley, R., Russell, S. G., Larkins, S., Taylor, S., Sagigi, B., Strivens, E., & Redman-MacLaren, M. (2022). Aging well for indigenous peoples: A scoping review. *Frontiers in Public Health*, *10*, 780898. 10.3389/fpubh.2022.780898 35223727 10.3389/fpubh.2022.780898 PMC8866315

[CR51] Rogers, C. (2021). Inclusion or exclusion: UK education policy and Roma pupils. In O. M. M.Mendes, & S. Tomia (Eds.), *Social and economic vulnerability of Roma people* (pp. 3–18). Springer.

[CR52] Sarafian, I., Robinson, A., Christov, A., & Tarchini, A. (2024). In the margins of stigma: Health inequalities among Bulgarian Roma in a post-COVID-19 UK. *BMJ Global Health*, *9*(11), e015686. 10.1136/bmjgh-2024-015686 10.1136/bmjgh-2024-015686 PMC11535758 39496362

[CR53] Scullion, L., & Brown, P. (2016). Understanding the social exclusion of Roma. In A. Ahmed, & M. Rogers (Eds.), *Working with Marginalised Groups: From Policy to Practice* (pp. 70–85). Palgrave Macmillan.

[CR54] Sixsmith, J., Sixsmith, A., Fänge, A. M., Naumann, D., Kucsera, C., Tomsone, S., Haak, M., Dahlin-Ivanoff, S., & Woolrych, R. (2014). Healthy ageing and home: the perspectives of very old people in five European countries. *Social science & medicine (1982)*, *106*, 1–9. 10.1016/j.socscimed.2014.01.006 24524960 10.1016/j.socscimed.2014.01.006

[CR55] Soja, E. W. (2010). *Seeking spatial justice*. University of Minnesota Press.

[CR56] Szilvasi, M., & Saitovic-Jovanovic, M. (2023). Social accountability and legal empowerment initiatives: Improving the health of underserved Roma communities in Eastern Europe. *Health and Human Rights*, *25*(1), 67. PMC9973508 37266311

[CR57] Valero, D., Elboj, C., Plaja, T., & Munté Pascual, A. (2021). Social work and the Roma community: Elements to improve current practices. *European Journal of Social Work, 24*(6), 978–989. 10.1080/13691457.2020.1857705

[CR58] Waugh, E., & Mackenzie, L. (2011). Ageing well from an urban Indigenous Australian perspective. *Australian Occupational Therapy Journal*, *58*(1), 25–33. 10.1111/j.1440-1630.2010.00914.x 21255029 10.1111/j.1440-1630.2010.00914.x

[CR59] WHO. (2019). *Roma health mediation in Romania: A case study*. WHO Regional Office for Europe.

[CR60] WHO (2014). *Pécs Declaration on Healthy Ageing for Roma Communities*. Retrieved June 4, 2025, from https://iris.who.int/handle/10665/365411#:~:text=The%20experts%20adopted%20the%20P%C3%A9cs,improving%20health%20among%20Roma%20communities

[CR61] WHO (2020). *Decade of Healthy Ageing 2020–2030*. World Health Organization. Retrieved June 4, 2025, from https://cdn.who.int/media/docs/default-source/decade-of-healthy-ageing/decade-proposal-final-apr2020-en.pdf

[CR62] Wiles, J. L., Leibing, A., Guberman, N., Reeve, J., & Allen, R. E. (2012). The meaning of aging in place to older people. *The Gerontologist*, *52*(3), 357–366. 10.1093/geront/gnr098 21983126 10.1093/geront/gnr098

[CR63] Woolrych, R., Sixsmith, J., Fisher, J., Makita, M., Lawthom, R., & Murray, M. (2021). Constructing and negotiating social participation in old age: experiences of older adults living in urban environments in the United Kingdom. *Ageing & Society*, *41*(6), 1398–1420. 10.1017/S0144686X19001569

[CR64] Woolrych, R., Sixsmith, J., Duvvuru, J., Portella, A., Fang, M. L., Menezes, D., & Lawthom, R. (2022). Cross-national perspectives on aging and place: implications for age-friendly cities and communities. *The Gerontologist*, *62*(1), 119–129. 10.1093/geront/gnab170 34791252 10.1093/geront/gnab170

[CR65] y Blasco, P. G., & Fotta, M. (2023). *Romani chronicles of COVID-19: Testimonies of harm and resilience*. Berghahn. 10.2307/jj.7079903

[CR66] Yarker, S., Doran, P., & Buffel, T. (2024). Theorizing “place” in aging in place: The need for territorial and relational perspectives. *The Gerontologist, 64*(2), gnad002. 10.1093/geront/gnad002 10.1093/geront/gnad002 PMC10809216 36655690

[CR67] Yazdanpanahi, M., & Woolrych, R. (2023). Neighbourhood environment, healthy aging, and social participation among ethnic minority adults over 50: The case of the Turkish-Speaking community in London. *Housing and Society*, *50*(2), 206–227. 10.1080/08882746.2022.2060010

[CR68] Zubair, M., & Norris, M. (2015). Perspectives on ageing, later life and ethnicity: ageing research in ethnic minority contexts. *Ageing & Society*, *35*(5), 897–916. 10.1017/S0144686X14001536 25937682 10.1017/S0144686X14001536 PMC4396438

